# Therapeutic Apheresis, Circulating PLD, and Mucocutaneous Toxicity: Our Clinical Experience through Four Years

**DOI:** 10.3390/pharmaceutics12100940

**Published:** 2020-09-30

**Authors:** Stanislav Filip, Ondřej Kubeček, Jiří Špaček, Miriam Lánská, Milan Bláha, Jiřina Martínková

**Affiliations:** 1 Department of Oncology and Radiotherapy, Faculty of Medicine in Hradec Králové, Charles University Prague, 50003 Hradec Králové, Czech Republic; ondrej.kubecek@fnhk.cz (O.K.); martinkova.jir@gmail.com (J.M.); 2 Department of Gynecology and Obstetrics, Faculty of Medicine in Hradec Králové, Charles University Prague, 50003 Hradec Králové, Czech Republic; jiri.spacek2@fnhk.cz; 3 4th Department of Internal Medicine-Haematology, Faculty Hospital in Hradec Králové, 50005 Hradec Králové, Czech Republic; miriam.lanska@fnhk.cz (M.L.); milan.blaha@fnhk.cz (M.B.)

**Keywords:** pegylated liposomal doxorubicin, chemotherapy, palmar-plantar erythrodysesthesia, therapeutic apheresis, chemotoxicity

## Abstract

Cancer treatment has been greatly improved by the combined use of targeted therapies and novel biotechnological methods. Regarding the former, pegylated liposomal doxorubicin (PLD) has a preferential accumulation within cancer tumors, thus having lower toxicity on healthy cells. PLD has been implemented in the targeted treatment of sarcoma, ovarian, breast, and lung cancer. In comparison with conventional doxorubicin, PLD has lower cardiotoxicity and hematotoxicity; however, PLD can induce mucositis and palmo-plantar erythrodysesthesia (PPE, hand-foot syndrome), which limits its use. Therapeutical apheresis is a clinically proven solution against early PLD toxicity without hindering the efficacy of the treatment. The present review summarizes the pharmacokinetics and pharmacodynamics of PLD and the beneficial effects of extracorporeal apheresis on the incidence of PPE during chemoradiotherapy in cancer patients.

## 1. Introduction

As a result of their non-selective effects, the use of conventional cytostatics is often influenced by dose-limiting toxicity, the point in which it affects both the tumor and normal cells. Therefore, a relatively narrow dosing range is necessary to both prevent such adverse/toxic effects and maintain its antitumor activity.

**Figure 1:**
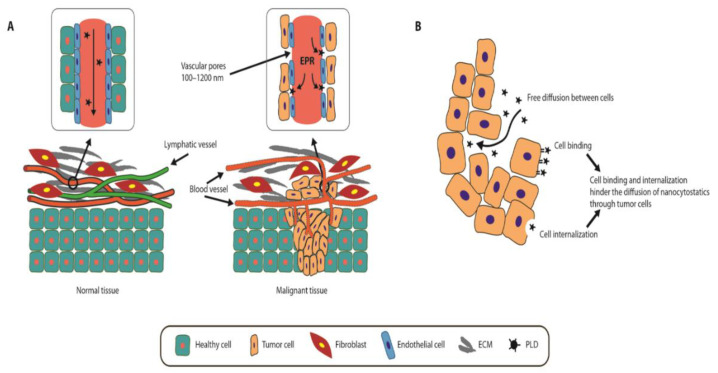
Targeted distribution of nanoparticles and EPR effect in tumors. (**A**)—PLD transport through solid tumors. Compared to normal tissues, tumors have a disorganized microvascular network with fenestrated capillaries and often lack lymphatic vessels. Such pathological covariates result in enhanced permeability and retention (EPR). ECM—extracellular matrix; EPR—enhanced permeability and retention; PLD—pegylated doxorubicin. **(B**)—EPR effect in tumors. PLD is distributed through tumors by diffusing freely in the extracellular microenvironment, after which it can be transported by cell binding and/or internalization. Some of the mechanisms regulating this transport include molecular diffusion, tumor interstitium hindrance, cell density, cell binding affinity, internalization, and systemic clearance. Adapted from [7], Dove Medical Press, 2017.

A solution to this problem was found in targeted nanoparticle-based drug delivery systems (DDSs) [1,2,3], which allow the delivery of an active substance into the target tissue simply by altering the pharmacokinetics and toxicological profile of the parent drug [4,5]. This targeted distribution is based on both the enhanced permeation and retention effect (EPR) of tumors [6,7,8], an effect that has been known for more than 30 years [9]. Under physiological conditions, the endothelium and basement membrane of the blood vessels prevent the passage of nanoparticles into the adjacent tissue because the tight junctions between endothelial cells are too small for particles greater than 20 nm in diameter. In contrast, the angiogenic blood vessels in many solid tumors are pathologically disorganized and often possess pathological covariates, such as endothelial fenestrations, increased capillary permeability, and defective lymphatic drainage (Figure 1A,B), which lead to tumor hyperpermeability [6,10,11]. The EPR effect is influenced by tumor type and size; in this regard, pancreatic, colon, breast, and stomach cancers have a great capacity for nanoparticle accumulation. Tumor perfusion and inflammation, among others, can also influence this accumulation capacity [12].

Targeted distribution depends greatly upon the physicochemical properties of the nanoparticles in question. There are three essential parameters governing the vascular dynamics of these drug carriers: pair collision force, deformation-induced lift force, and shear gradient force, all of which are determined by its size, shape, stiffness, and surface functionality. These “4S” properties are strongly correlated with the “passive tumor-targeting” characteristics of a nanoparticle [13].

It is possible to reduce the toxicity of a drug by removing the excess nanoparticles from circulation. This can be achieved by therapeutical apheresis provided that the pharmacokinetics profile of these nanoparticles is reliable (see below). In our previous clinical study, we used a modified double plasma filtration (DFPP) protocol. The original method is performed in two steps, which involve conventional plasma filtration and a second therapeutic plasma filtration using special filters [14]. In our case, plasma filtration was first performed by centrifugation using a separator and later passed through a special filter, where the main therapeutic process was done [15]. The efficacy and safety of these methods were evaluated in solid tumor patients treated with pegylated liposomal doxorubicin. The present review presents an overview on the pharmacokinetics and pharmacodynamics of PLD as well as the beneficial effects of extracorporeal apheresis in the prevention of PPE during the treatment of cancer patients.

## 2. Pegylated Liposomal Doxorubicin (PLD)

Non-liposomal doxorubicin, also known as adriamycin, is an anthracycline antibiotic composed of a fluorescent tetracyclic chromophore (doxorubicione, the planar phenoxazone actinosine) linked to a positively charged amino sugar. The drug enters a cell through a flip-flop-based mechanism across the membrane [16], later entering into the nucleus through the nuclear pore complex. Doxorubicin was assumed to have minimal back diffusion from the tumor into peripheral blood, which was confirmed in an animal model [17]. Once in the nucleus, the planar phenoxazone ring of doxorubicin allows it to intercalate between adjacent guanine-cytosine base pairs in the cell’s DNA. The stability of this complex is sufficient to block its transcription by RNA polymerase II [18]. In addition, doxorubicin interacts with mitochondria through its affinity with cardiolipin and with intracellular iron, thus generating a high quantity of reactive oxygen species (ROS). The generation of ROS by doxorubicin is enhanced in cardiomyocytes by the presence of intracellular non-chelated iron [19]. Since cardiomyocytes are much more sensitive to oxidative stress, these free radicals are responsible for the unusual and often irreversible cardiotoxic effects of doxorubicin [20].

PLD, on the other hand, is a formulation of doxorubicin hydrochloride encapsulated in polyethylene glycol-coated stealth liposomes of 65–100 nm in diameter. The pharmacokinetic highlights of PLD are characterized by its dramatically reduced distribution system and its long period of elimination (clearance rate) [20]. Concerning the former, the major advantage of PLD lies in the efficient delivery and accumulation of doxorubicin within a solid tumor, i.e., kinetic targeting [8,21,22], which has been demonstrated in various animal studies [17,23]. However, the distribution of PLD also depends upon other pathological covariates; in some cases, PLD can be present in ascitic fluid and pleural effusion several days after drug administration, meaning that its systemic distribution is significantly increased [24,25]. Regarding its period of elimination, PLD is able to prevent the rapid clearance of doxorubicin by the reticuloendothelial system. The clearance of pegylated liposomes, and that of PLD, is determined by either its degradation, e.g., phagocyte uptake, aggregation, pH-sensitive breakdown, etc., or by content release [26]; in the latter case, the released doxorubicin is eliminated by the kidneys or by hepatic metabolism. Due to its therapeutic characteristics, PLD has been approved for the treatment of ovarian and breast cancer, multiple myeloma, and Kaposi sarcoma [27]. In patients with ovarian cancer, the use of PLD is only advantageous, concerning disease progression, in cases of “platinum-sensitive” disease and second-line treatment [24].

## 3. Side Effects of PLD Treatment

The toxicological profile of PLD is vastly different from that of non-liposomal doxorubicin. Due to the reduced distribution of polyethylenglycol-coated stealth liposomes, the toxicodynamic properties of doxorubicin are greatly diminished, resulting in significantly lower cardiotoxicity, milder myelosuppression, and a greatly reduced incidence of alopecia. Typically, the cumulative dose of doxorubicin is restricted to 450 mg/m^2^ whereas that of PLD can be as high as ≤900 mg/m^2^ [28].

However, the prolonged circulation time of PLD also results in the release of doxorubicin in healthy tissues. In this regard, the skin is the major site of liposome accumulation; therefore, PLD treatment could induce a significant liposome uptake and increase the risk of hand-foot syndrome (palmar-plantar erythrodysesthesia) [29]. This increased uptake has been ascribed to mucous tissue and membranes, thus resulting in dose-limiting serious events [29,30].

**Figure 2:**
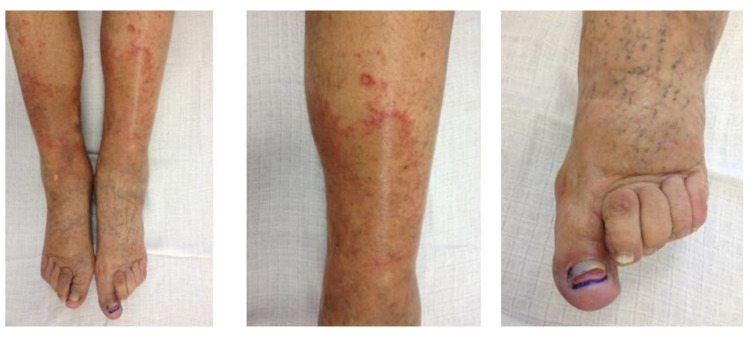
Palmar-plantar erythrodysesthesia (PPE or hand-foot syndrome). A 64-year-old patient with platinum-resistant ovarian cancer was included in the first Czech clinical study (EudraCT number 2016-000971-26). The study was approved by the Ethics Committee of Charles University and the Faculty of Medicine in Hradec Králové number 201506S30P and by the State Institute for Drug Control Czech Republic number SUKLS 74616-2016. The patient was treated with a 1-h IV infusion of 50 mg/m^2^ of PLD/cycle without plasma filtration. PPE (grade 3) was observed during the second cycle, which was treated with corticosteroids, antibiotics, and analgesics for three weeks.

The palmar-plantar erythrodysesthesia (PPE) syndrome culminates in painful erythema and swelling, especially in the areas commonly exposed to pressure, such as the hand palms and the soles of the feet, followed by skin desquamation and re-epithelization. The severity of PPE may range from mild erythema to severe skin damage causing temporary invalidity to the patient (Figure 2). It must be mentioned that this skin affection is temporary, and the patients show a complete restitution after 2–3 weeks [21,31]. Dosage reduction seems to have little impact on the occurrence of PPE.

On the other hand, mucositis usually occurs in the form of stomatitis, although rare cases of pharyngo-esophagitis and vulvo-vaginitis have also been reported [32]. The incidence of mucositis has been strongly associated with the amount of PLD administered in a single dose, unlike PPE, which is instead associated with continuous dosing, i.e., dose/time interval between dose administration [33,34]. PPE and mucositis are the most common adverse effects of PLD treatment (Table 1).

## 4. Therapeutic Removal of PLD through Double Plasma Filtration (DFPP)

**Figure 3:**
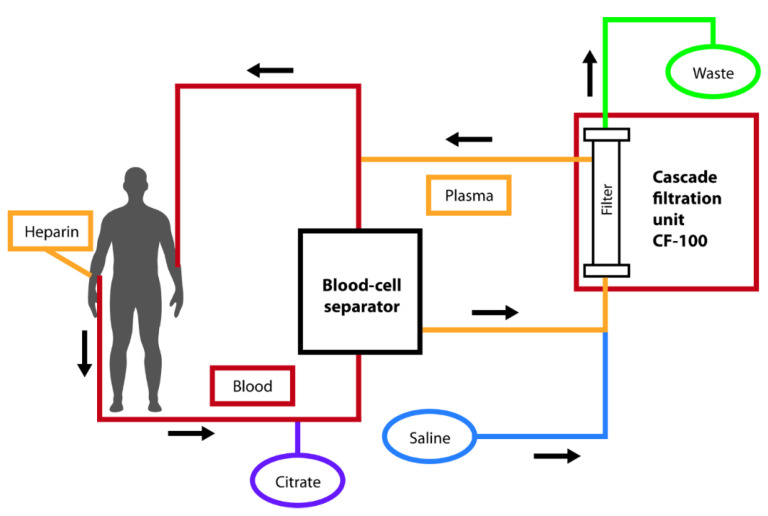
DFPP as modified by our group (using a separator). The modified method was performed in the following manner: Cell-free plasma was obtained by centrifuging the samples in a quality separator (Cobe-Spectra or Optia, Terumo, Likewood, OA, USA) and filtered through an Evaflux 4A filter (Kawasumi, Tokio, Japan) with ethylene-vinyl alcohol hollow fibers (pore diameter of 0.03 µm). Both the plasma flow and volume were calculated by the computer included in the instrument. Anticoagulation: ACD-A (Baxter, Munchen, Germany) 1:15 and a basic bolus of 4000 units of heparin i.v. were administered [39]. (1) Red arrow—peripheral blood collection; (2) Blue arrow—plasma collection; (3) Green arrow—waste. (4) Black arrow—the blood-cell separator. (5) Orange arrow—application of heparin and analysis of plasma.

Once a tumor becomes saturated with doxorubicin, most of the administered dose remains in circulation in the plasma, contributing to the toxicity observed in peripheral tissues. The antitumor activity and toxicity of PLD is often credited to its pharmacokinetics [30,33]. Regarding the former, its optimum time of exposure is estimated between 48 and 72 h post infusion, when the maximum concentration is achieved in the tumor (C_Tmax_) [24]. It is believed that the spontaneous release of the cytostatic agent from the pegylated liposomes in circulation (leakage) could contribute to toxicity [38]. A previous study confirmed a maximum 10% exposure to non-liposomal doxorubicin under a PLD treatment, which is significantly lower in comparison with conventional doxorubicin treatments [25]. After reaching C_Tmax_ in the tumor, the ideal scenario would be to achieve the complete removal of circulating PLD. To such end, DFPP following plasma segregation has been found to be an effective and safe method in this regard [14,22,39]. This strategy enables the efficient extracorporeal removal of circulating PLD by promoting its low extent of distribution, negligible plasma protein binding, and a prolonged drug elimination rate. DFPP and the modifications used in our clinical study have two main steps. In the first, plasma filtration is performed using a separator whereas that in the second step PLD is eliminated using a special filter (Figure 3) [39].

Through the implementation of DFPP, the CARL trial was the first study to reduce PLD toxicity in patients suffering from breast cancer [14,22]. In that study, PLD was administered in doses of 40 mg/m^2^ every 3 weeks in combination with 2 × 25 mg/m^2^ vinorelbine (neoadjuvant treatment of breast cancer, 12 patients) or as 40 mg/m^2^ every 4 weeks (recurrent ovarian cancer, 3 patients). The primary endpoints of the study were to evaluate the efficiency and safety profile of DFPP, whereas its secondary endpoints tried to determine its toxicity and tumor response. As a result, it was found that DFPP scheduled at 44–48 h post dose decreased PLD exposure by 62% and no DFPP-related toxicity was observed. Only five grade 2 events and one grade 3 event of mucositis, neutropenia, or leucopenia were observed, and a single grade 2 palmar-plantar erythrodysesthesia case was reported in relation to the PLD treatment. No spontaneous leakage was described. Further, a >30% reduction in tumor size occurred in 10/12 (neoadjuvant) and in 1/3 (recurrent) patients.

In the Czech clinical study [15,39,40], a modified plasma filtration protocol was used in patients with platinum-resistant ovarian cancer, proving that its PLD removal efficiency was rather high [40]. The project EudraCT number 2016-000971-26 was approved by the Ethics Committee of Charles University and the Faculty of Medicine in Hradec Králové number 201506S30P and by the State Institute for Drug Control Czech Republic number SUKLS 74616-2016. The efficiency of 2–3 h plasma filtration PF scheduled at 44–46 h post dose was considered high enough regarding the removal of PLD (a standard dose 50 mg/m^2^ in 1-h IV infusion every 28 days at the beginning of each chemotherapy cycle). Further, this study was the first to estimate the individual native of the pharmacokinetics of PLD prior to plasma filtration, accounting for both independent and plasma filtration-related variables, determining that the mean plasma filtration clearance rate was 42-fold more efficient when compared to its native clearance. Although the PLD fraction eliminated in vivo is comparable to that eliminated by plasma filtration: 31% (10) vs. 34% (7) [25], it is significantly longer (44–46 h) when compared to PF (2–3 h). Regarding PF-related adverse effects, only one case of grade 3 PPE was observed after a second PLD dose, leading to the interruption of the treatment. The occurrence of only one case in this regard is in contrast with previous phase III trial reports, which showed an incidence as high as 50% [36]. The risk of PPE can be significantly higher in the presence of comorbid pathologies, such as cutaneous reflux, increased lymphatic vessel permeability, and the availability of collecting channels, all of which can be observed in severe venous insufficiency patients. Throughout our clinical experience, we have observed few instances of adverse effects in relation with plasma filtration (7%). In contrast, median progression-free survival (PFS) was 3.6 months (1.5–8.1) whereas overall survival (OS) was 7.5 months (1.7–26.7), and 33% of the treated patients achieved stable disease and 62% displayed disease progression.

However, it is difficult to compare the previous CARL trial with our Czech study. The patients with breast cancer involved in the former were treated with a combined PLD + vinorelbine therapy, while the patients from the latter were under ovarian cancer monotherapy. From a methodological standpoint, we can prove that our DFPP modified method is clinically safe and has little to no adverse effects. Moreover, DFPP seems to be more efficient in removing PLD from the circulation than other methods excluding the use of a separator, although no relevant clinical data from the antitumor activity in the CARL trial therapy is available to make a proper comparison. This method is not without its shortcoming, though, as the efficient removal of PLD depends upon the technical equipment of the workplace and the staff’s experience with therapeutic plasma filtration.

## 5. Future Perspectives

Excess PLD can be removed without affecting its overall concentration in the tumor or hindering its antitumor activity provided that plasma filtration is appropriately scheduled [23]. Therefore, correct PLD dosing and plasmapheresis timing is crucial for an effective and safe treatment, as demonstrated in a previous animal study [23]. In such a report, plasmapheresis scheduled 24 h after the administration of PLD interfered with its therapeutic efficacy, whereas when applied at 36 h post administration, the antitumor efficacy of PLD could be preserved with the added benefit of reduced doxorubicin toxicity [23].

Ideally, future therapeutic strategies could implement plasma filtration and postpone its application until 72 h after PLD administration to improve its antitumor activity. Moreover, the dose fraction removed during the previous cycle could be supplemented in the next one. To optimize the treatment’s timing and strategy, mathematical modeling could also be implemented to determine the pharmacokinetics and dynamics of the therapeutic drugs [41,42]. In this regard, recent mathematical models have described the dynamics of the tumor’s size following anticancer drug treatment [43].

Anthracyclines are very effective radiosensitizers [44,45], i.e., PLD and radiation therapy result in a greater antitumor effect for the latter. However, the concurrent use of radiotherapy and anthracycline-based chemotherapy has a considerably high toxicity, thus limiting its use [46]. Further, a recall phenomenon has been reported for this group of cytostatics due to an acute inflammatory reaction induced at previously irradiated sites [47,48]; such an effect can be observed after months or years of chemotherapy completion [47,49].

Achieving a higher concentration of anthracyclines within the tumor while diminishing its accumulation in healthy or sensitive tissues by means of plasma filtration would very likely result in a significantly greater antitumor effect and lower radiotoxicity. The combination of both treatments seems particularly advantageous against tumors sensitive to both radiotherapy and anthracyclines, such as breast carcinomas or sarcomas. In addition to radiotherapy, it may be possible to combine the therapeutic removal of excess circulating drugs with other modalities, such as hyperthermia [50,51,52], that can increase the effectiveness of chemotherapy through thermolabile liposomes, e.g., hyperthermia increases both PLD extravasation and doxorubicin release from liposomes in animal models [53,54]. While the advances made in clinical radiotherapy have driven many technological innovations, continuous improvement relies on the rational combination of different therapeutic strategies. Undeniably, the improved understanding of cancer biology will propel the field of radiotherapy forward by allowing the integration of novel nanotechnology-based treatments [55,56].

## Figures and Tables

**Table 1:** PLD toxicity as observed in our clinical trials.

Toxicity	Gordon et al. (2000) [35] n = 89 *	Gordon et al. (2001) [36] n = 239 *	Colombo et al. (2012) [37] n = 409 *	Kubeček et al. (2020) [15] n = 15 **
Anemia (Any Grade)	35 (39.3%)	85 (35.6%)	88 (21.5%)	5 (33.3%)
Grade 3 to 4	12 (13.5%)	13 (5.4%)	15 (3.7%)	0
Thrombocytopenia (Any Grade)	n/a	31 (13.0%)	n/a	2 (13.3%)
Grade 3 to 4	n/a	3 (1.3%)	n/a	0
Neutropenia (Any Grade)	33 (37.1%)	84 (35.1%)	89 (21.8%)	0
Grade 3 to 4	14 (15.7%)	29 (12.1%)	41 (10.0%)	0
PPE (Hand-foot syndrome)	37 (41.6%)	117 (49.0%)	171 (41.8%)	1 (6.7%)
Grade 3 to 4	18 (20.2%)	55 (23.0%)	55 (13.4%)	1 (6.7%)
Mucositis (Any Grade)	31 (34.8%)	95 (39.7%)	176 (43.0%)	1 (6.7%)
Grade 3 to 4	8 (9.0%)	20 (8.4%)	41 (10.0%)	0
Nausea (Any Grade)	34 (38.2%)	n/a	188 (46.0%)	6 (40.0%)
Grade 3 to 4	6 (6.7%)	n/a	24 (5.9%)	1 (6.7%)
Vomiting (Any Grade)	17 (19.1%)	n/a	131 (32.0%)	4 (26.7%)
Grade 3 to 4	4 (4.5%)	n/a	24 (5.9%)	0
Constipation (Any Grade)	n/a	n/a	143 (35.0%)	3 (20.0%)
Grade 3 to 4	n/a	n/a	11 (2.7%)	0
Fatigue (Any Grade)	37 (41.6%)	n/a	190 (46.5%)	2 (13.3%)
Grade 3 to 4	8 (9.0%)	n/a	34 (8.3%)	0

* Without plasma filtration. ** With plasma filtration.

## References

[1] Pütz G., Schmah O., Eckes J., Hug M.J., Winkler K. (2009). Controlled application and scheduled removal of nanoparticle based chemotherapeutics (CARL) will reduce dose limiting adverse events in anticancer chemotherapy. Med. Hypotheses.

[2] Waite C.L., Roth C.M. (2012). Nanoscale drug delivery systems for enhanced drug penetration into solid tumors: Current progress and opportunities. Crit. Rev. Biomed. Eng..

[3] Zhang R.X., Ahmed T., Li L.Y., Li J., Abbasi A.Z., Wu X.Y. (2017). Design of nanocarriers for nanoscale drug delivery to enhance cancer treatment using hybrid polymer and lipid building blocks. Nanoscale.

[4] Allen T.M., Cullis P.R. (2004). Drug Delivery Systems: Entering the Mainstream. Science.

[5] Samad A., Sultana Y., Aqil M. (2007). Liposomal drug delivery systems: An update review. Curr. Drug Deliv..

[6] Fang J., Nakamura H., Maeda H. (2011). The EPR effect: Unique features of tumor blood vessels for drug delivery, factors involved, and limitations and augmentation of the effect. Adv. Drug Deliv. Rev..

[7] Millard M., Yakavets I., Zorin V., Kulmukhamedova A., Marchal S., Bezdetnaya L. (2017). Drug delivery to solid tumors: The predictive value of the multicellular tumor spheroid model for nanomedicine screening. Int. J. Nanomed..

[8] Gabizon A., Shmeeda H., Barenholz Y. (2003). Pharmacokinetics of Pegylated Liposomal Doxorubicin. Clin. Pharmacokinet..

[9] Matsumura Y., Maeda H. (1986). A new concept for macromolecular therapeutics in cancer chemotherapy: Mechanism of tumoritropic accumulation of proteins and the antitumor agent smancs. Cancer Res..

[10] Maeda H., Wu J., Sawa T., Matsumura Y., Hori K. (2000). Tumor vascular permeability and the EPR effect in macromolecular therapeutics: A review. J. Control. Release.

[11] Maeda H. (2017). Polymer therapeutics and the EPR effect. J. Drug Target..

[12] Natfji A.A., Ravishankar D., Osborn H.M.I., Greco F. (2017). Parameters Affecting the Enhanced Permeability and Retention Effect: The Need for Patient Selection. J. Pharm. Sci..

[13] Ye H., Shen Z., Li Y., Wei M., Li Y. (2018). Manipulating nanoparticle transport within blood flow through external forces: An exemplar of mechanics in nanomedicine. Proc. Math. Phys. Eng. Sci..

[14] Pütz G., Schmah O., Eckes J., Hug M.J., Winkler K. (2010). Controlled application and removal of liposomal therapeutics: Effective elimination of pegylated liposomal doxorubicin by double-filtration plasmapheresis in vitro. J. Clin. Apher..

[15] Kubecek O., Martínková J., Chladek J., Bláha M., Maláková J., Hodek M., Špaček J., Filip S. (2019). Plasmafiltration as an effective method in the removal of circulating pegylated liposomal doxorubicin (PLD) and the reduction of mucocutaneous toxicity during the treatment of advanced platinum-resistant ovarian cancer. Cancer Chemother. Pharmacol..

[16] Regev R., Yeheskely-Hayon D., Katzir H., Eytan G.D. (2005). Transport of anthracyclines and mitoxantrone across membranes by a flip-flop mechanism. Biochem. Pharmacol..

[17] Ngoune R., Peters A., Von Elverfeldt D., Winkler K., Pütz G. (2016). Accumulating nanoparticles by EPR: A route of no return. J. Control Release.

[18] Yang F., Kemp C.J., Henikoff S. (2015). Anthracyclines induce double-strand DNA breaks at active gene promoters. Mutat. Res. Mol. Mech. Mutagen..

[19] Kotamraju S., Kalivendi S.V., Konorev E., Chitambar C.R., Joseph J., Kalyanaraman B. (2004). Oxidant-Induced Iron Signaling in Doxorubicin-Mediated Apoptosis. Enzyme Engineering and Evolution: General Methods.

[20] Minotti G., Menna P., Salvatorelli E., Cairo G., Gianni L. (2004). Anthracyclines: Molecular Advances and Pharmacologic Developments in Antitumor Activity and Cardiotoxicity. Pharmacol. Rev..

[21] Gabizon A.A. (2001). Pegylated Liposomal Doxorubicin: Metamorphosis of an Old Drug into a New Form of Chemotherapy. Cancer Investig..

[22] Eckes J., Schmah O., Siebers J.W., Groh U., Zschiedrich S., Rautenberg B., Hasenburg A., Jansen M., Hug M.J., Winkler K. (2011). Kinetic Targeting of pegylated liposomal Doxorubicin: A new Approach to Reduce Toxicity during Chemotherapy (CARL-trial). BMC Cancer.

[23] Ngoune R., Contini C., Hoffmann M.M., Von Elverfeldt D., Winkler K., Putz G. (2018). Optimizing Antitumor Efficacy and Adverse Effects of Pegylated Liposomal Doxorubicin by Scheduled Plasmapheresis: Impact of Timing and Dosing. Curr. Drug Deliv..

[24] Gabizon A.A., Patil Y., La-Beck N.M. (2016). New insights and evolving role of pegylated liposomal doxorubicin in cancer therapy. Drug Resist. Updates.

[25] Tahover E., Patil Y.P., Gabizon A.A. (2015). Emerging delivery systems to reduce doxorubicin cardiotoxicity and improve therapeutic index. Anti-Cancer Drugs.

[26] Ichihara M., Shimizu T., Imoto A., Hashiguchi Y., Uehara Y., Ishida T., Kiwada H. (2010). Anti-PEG IgM Response against PEGylated Liposomes in Mice and Rats. Pharmaceutics.

[27] Sousa I., Rodrigues F., Prazeres H., Lima R.T., Soares P. (2018). Liposomal therapies in oncology: Does one size fit all?. Cancer Chemother. Pharmacol..

[28] O’Brien M.E.R., Wigler N., Inbar M., Rosso R., Grischke E., Santoro A., Catane R., Kieback D.G., Tomczak P., Ackland S.P. (2004). Reduced cardiotoxicity and comparable efficacy in a phase IIItrial of pegylated liposomal doxorubicin HCl(CAELYX™/Doxil^®^) versus conventional doxorubicin forfirst-line treatment of metastatic breast cancer. Ann. Oncol..

[29] Gandy J., How C., Harrold K. (2007). Palmar–plantar erythrodysesthesia (PPE): A literature review with commentary on experience in a cancer centre. Eur. J. Oncol. Nurs..

[30] Boers-Sonderen M.J., Van Herpen C.M.L., Van Der Graaf W.T.A., Desar I.M.E., Van Der Logt M.G.W.A., De Beer Y.M., Ottevanger P.B., Van Erp N.P. (2014). Correlation of toxicity and efficacy with pharmacokinetics (PK) of pegylated liposomal doxorubicin (PLD) (Caelyx^®^). Cancer Chemother. Pharmacol..

[31] Bun S., Yunokawa M., Tamaki Y., Shimomura A., Shimoi T., Kodaira M., Shimizu C., Yonemori K., Fujiwara Y., Makino Y. (2018). Symptom management: The utility of regional cooling for hand-foot syndrome induced by pegylated liposomal doxorubicin in ovarian cancer. Support. Care Cancer.

[32] Solomon R., Gabizon A.A. (2008). Clinical Pharmacology of Liposomal Anthracyclines: Focus on Pegylated Liposomal Doxorubicin. Clin. Lymphoma Myeloma.

[33] Lyass O., Uziely B., Ben-Yosef R., Tzemach D., Heshing N.I., Lotem M., Brufman G., Gabizon A. (2000). Correlation of toxicity with pharmacokinetics of pegylated liposomal doxorubicin (Doxil) in metastatic breast carcinoma. Cancer.

[34] Minisini A.M., Andreetta C., Fasola G., Puglisi F. (2008). Pegylated liposomal doxorubicin in elderly patients with metastatic breast cancer. Expert Rev. Anticancer. Ther..

[35] Gordon A.N., Granai C., Rose P.G., Hainsworth J., Lopez A., Weissman C., Rosales R., Sharpington T. (2000). Phase II Study of Liposomal Doxorubicin in Platinum- and Paclitaxel-Refractory Epithelial Ovarian Cancer. J. Clin. Oncol..

[36] Gordon A.N., Fleagle J.T., Guthrie D., Parkin D.E., Gore M.E., Lacave A.J. (2001). Recurrent Epithelial Ovarian Carcinoma: A Randomized Phase III Study of Pegylated Liposomal Doxorubicin Versus Topotecan. J. Clin. Oncol..

[37] Colombo N., Kutarska E., Dimopoulos M., Bae D.-S., Rzepka-Gorska I., Bidzinski M., Scambia G., Engelholm S.A., Joly F., Weber D. (2012). Randomized, Open-Label, Phase III Study Comparing Patupilone (EPO906) With Pegylated Liposomal Doxorubicin in Platinum-Refractory or -Resistant Patients With Recurrent Epithelial Ovarian, Primary Fallopian Tube, or Primary Peritoneal Cancer. J. Clin. Oncol..

[38] Mayer L.D., Tai L.C., Ko D.S., Masin D., Ginsberg R.S., Cullis P.R., Bally M.B. (1989). Influence of vesicle size, lipid composition, and drug-to-lipid ratio on the biological activity of liposomal doxorubicin in mice. Cancer Res..

[39] Blaha M., Martinkova J., Lanska M., Filip S., Malakova J., Kubecek O., Bezouška J., Spacek J. (2017). Plasma filtration for the controlled removal of liposomal therapeutics—From the apheretic site of view. Atheroscler. Suppl..

[40] Martinkova J., Bláha M., Kubecek O., Malakova J., Spacek J., Bezouška J., Krulichová I.S., Filip S. (2015). Plasmafiltration as a possible contributor to kinetic targeting of pegylated liposomal doxorubicin (PLD) in order to prevent organ toxicity and immunosuppression. Cancer Chemother. Pharmacol..

[41] Moss D.M., Siccardi M. (2014). Optimizing nanomedicine pharmacokinetics using physiologically based pharmacokinetics modelling. Br. J. Pharmacol..

[42] Schultink A.H.M.D.V., Suleiman A.A., Schellens J.H.M., Beijnen J.H., Huitema A.D.R. (2016). Pharmacodynamic modeling of adverse effects of anti-cancer drug treatment. Eur. J. Clin. Pharmacol..

[43] Ribba B., Holford N., Magni P., Trocóniz I.F., Gueorguieva I., Girard P., Sarr C., Elishmereni M., Kloft C., Friberg L.E. (2014). A Review of Mixed-Effects Models of Tumor Growth and Effects of Anticancer Drug Treatment Used in Population Analysis. CPT: Pharmacometrics Syst. Pharmacol..

[44] Jagetia G.C., Nayak V. (2000). Effect of doxorubicin on cell survival and micronuclei formation in HeLa cells exposed to different doses of gamma-radiation. Strahlenther. Onkol..

[45] Wu S., Chou H., Yuh C., Mekuria S.L., Kao Y., Tsai H.-C. (2017). Radiation-Sensitive Dendrimer-Based Drug Delivery System. Adv. Sci..

[46] Fiets W., Van Helvoirt R., Nortier J., Van Der Tweel I., Struikmans H. (2003). Acute toxicity of concurrent adjuvant radiotherapy and chemotherapy (CMF or AC) in breast cancer patients. Eur. J. Cancer.

[47] Bahaj W., Ya’Qoub L., Toor M., Masood A. (2019). Radiation Recall in a Patient with Intrahepatic Cholangiocarcinoma: Case Report and a Literature Review. Cureus.

[48] Burris H.A., Hurtig J. (2010). Radiation Recall with Anticancer Agents. Oncologist.

[49] Camidge R., Price A. (2001). Characterizing the phenomenon of radiation recall dermatitis. Radiother. Oncol..

[50] Wei Q., Xu W.-H., Han M., Dong Q., Fu Z.-X., Diao Y.-Y., Liu H., Xu J., Jiang H.-L., Zheng S. (2012). Doxorubicin-mediated radiosensitivity in multicellular spheroids from a lung cancer cell line is enhanced by composite micelle encapsulation. Int. J. Nanomed..

[51] Dicheva B.M., Koning G.A. (2013). Targeted thermosensitive liposomes: An attractive novel approach for increased drug delivery to solid tumors. Expert Opin. Drug Deliv..

[52] Lokerse W.J., Bolkestein M., Hagen T.L.T., De Jong M., Eggermont A.M., Grüll H., Koning G.A. (2016). Investigation of Particle Accumulation, Chemosensitivity and Thermosensitivity for Effective Solid Tumor Therapy Using Thermosensitive Liposomes and Hyperthermia. Theranostics.

[53] Huang S.K., Stauffer P.R., Hong K., Guo J.W., Phillips T.L., Huang A., Papahadjopoulos D. (1994). Liposomes and hyperthermia in mice: Increased tumor uptake and therapeutic efficacy of doxorubicin in sterically stabilized liposomes. Cancer Res..

[54] Willerding L., Limmer S., Hossann M., Zengerle A., Wachholz K., Hagen T.L.T., Koning G.A., Sroka R., Lindner L.H., Peller M. (2016). Method of hyperthermia and tumor size influence effectiveness of doxorubicin release from thermosensitive liposomes in experimental tumors. J. Control. Release.

[55] Mi Y., Shao Z., Vang J., Kaidar-Person O., Wang A.Z. (2016). Application of nanotechnology to cancer radiotherapy. Cancer Nanotechnol..

[56] DuRoss A.N., Neufeld M.J., Rana S., Thomas C.R., Sun C. (2019). Integrating nanomedicine into clinical radiotherapy regimens. Adv. Drug Deliv. Rev..

