# Erratum: Filip, S.; et al. Therapeutic Apheresis, Circulating PLD, and Mucocutaneous Toxicity: Our Clinical Experience through Four Years. *Pharmaceutics* 2020, *12*, 940

**DOI:** 10.3390/pharmaceutics12111103

**Published:** 2020-11-17

**Authors:** Stanislav Filip, Ondřej Kubeček, Jiří Špaček, Miriam Lánská, Milan Bláha

**Affiliations:** 1 Department of Oncology and Radiotherapy, Faculty of Medicine in Hradec Králové, Charles University Prague, 50003 Hradec Králové, Czech Republic; ondrej.kubecek@fnhk.cz; 2 Department of Gynecology and Obstetrics, Faculty of Medicine in Hradec Králové, Charles University Prague, 50003 Hradec Králové, Czech Republic; jiri.spacek2@fnhk.cz; 3 4th Department of Internal Medicine-Haematology, Faculty Hospital in Hradec Králové, 50005 Hradec Králové, Czech Republic; miriam.lanska@fnhk.cz (M.L.); milan.blaha@fnhk.cz (M.B.)

The following correction has been made to this paper [1]: We would like to inform the readers that our colleague, Jirina Martinkova, asked to be excluded as an author in this communication. Although we regret her decision, we have respectfully fulfilled her request and wish her the best in her future endeavors. All authors accepted this change and signed Change of Authorship Form.
